# Improving Cardiac Reprogramming for Heart Regeneration in Translational Medicine

**DOI:** 10.3390/cells10123297

**Published:** 2021-11-25

**Authors:** Liu Liu, Yijing Guo, Zhaokai Li, Zhong Wang

**Affiliations:** 1 Department of Cardiac Surgery, Cardiovascular Center, The University of Michigan, Ann Arbor, MI 48109, USA; luvul@med.umich.edu (L.L.); yijing@umich.edu (Y.G.); lizhaokai@csu.edu.cn (Z.L.); 2 Department of Cardiology, Shanghai Jiao Tong University Affiliated Sixth People’s Hospital, Shanghai 200233, China; 3 Department of Cardiovascular Medicine, Xiangya Hospital, Central South University, Changsha 410000, China

**Keywords:** cardiac reprogramming, heart regeneration, cardiogenesis, pathological microenvironment, inflammation, pro-fibrotic signaling, nanoparticle, mechanical property

## Abstract

Direct reprogramming of fibroblasts into CM-like cells has emerged as an attractive strategy to generate induced CMs (iCMs) in heart regeneration. However, low conversion rate, poor purity, and the lack of precise conversion of iCMs are still present as significant challenges. In this review, we summarize the recent development in understanding the molecular mechanisms of cardiac reprogramming with various strategies to achieve more efficient iCMs. reprogramming. Specifically, we focus on the identified critical roles of transcriptional regulation, epigenetic modification, signaling pathways from the cellular microenvironment, and cell cycling regulation in cardiac reprogramming. We also discuss the progress in delivery system optimization and cardiac reprogramming in human cells related to preclinical applications. We anticipate that this will translate cardiac reprogramming-based heart therapy into clinical applications. In addition to optimizing the cardiogenesis related transcriptional regulation and signaling pathways, an important strategy is to modulate the pathological microenvironment associated with heart injury, including inflammation, pro-fibrotic signaling pathways, and the mechanical properties of the damaged myocardium. We are optimistic that cardiac reprogramming will provide a powerful therapy in heart regenerative medicine.

## 1. Introduction

Inspired by the induced pluripotent stem cells (iPSCs) reported by Takahashi et al. [1], converting one type of somatic cells into another became an important strategy in regenerative medicine. Researchers have attempted to convert fibroblasts into cardiomyocyte-like cells to regenerate cardiac tissue to compensate for fibrosis that forms after myocardial infarction. In 2010, Ieda et al. reported the introduction of three developmental transcriptional factor reprograms, post-natal cardiac or dermal fibroblasts, into induced cardiomyocytes (iCMs) [2]. The screening of 14 transcription factors and epigenetic remodeling factors, by comparing gene expression levels between mouse cardiomyocytes and cardiac fibroblasts, has enabled them to identify that Gata4, Mef2c, and Tbx5 (GMT) are sufficient for cardiac reprogramming. After infecting fibroblasts with retroviruses, or lentiviruses encoding three specific transcriptional factors, nearly 30% of cardiac fibroblast-derived iCMs exhibit spontaneous Ca^2+^ oscillations and a portion of them showed spontaneous contraction.

Though GMT-induced cardiac reprogramming is able to transdifferentiate part of fibroblasts into iCMs, low efficiency remains a significant challenge for future clinical translation. In addition, iCMs induced by GMT in vitro are still very different from mature cardiomyocytes in terms of molecular and electrophysiological phenotypes [3]. Therefore, in order to improve cardiac reprogramming efficiency, researchers have explored cardiac reprogramming mechanisms regulated by transcription, signaling pathways/kinases, epigenetics, and cell cycles. In this review, we summarize the recent understanding of cardiac reprogramming in mouse cells, as well as the progress of cardiac reprogramming in human cells and in vivo. We are highly optimistic that iCM reprogramming-based heart regeneration therapy will restore the pumping function of damaged hearts.

## 2. Transcriptional Regulation in Cardiac Reprogramming

### 2.1. Additional Transcriptional Factors Screening

Different transcriptional factor combinations or additional transcriptional factors based on GMT, have been examined to improve cardiac reprogramming efficiency. In one transcription factor screen experiment, iCMs induced by the combination of Tbx5, Mef2c and Myocd show higher cardiomyocyte marker gene expression than GMT-induced iCMs by Q-PCR assay [4]. It has also been identified that GMT and Hand2 (GHMT) can cooperatively reprogram adult mouse tail-tips and cardiac fibroblasts into iCMs, which show a higher percentage of α-MHC-GFP+, cTnT+, and α-MHC-GFP/cTnT+ cells compared to GMT-induced iCMs [5]. Another study shows that the overexpression of transcription factors MYOCD and SRF, alone or in conjunction with Mesp1 and SMARCD3, enhances the cardio-inducing effect of GMT in cardiac reprogramming by improving global cardiac related gene expression and corresponding cardiac specific functions including Ca^2+^ transient oscillations [6]. Apart from α-MHC-GFP+ fibroblasts, a screening platform that uses calcium transient as the major measurement of reprogramming has been established [7]. In this study, the combination of Hand2, Nkx2.5, Gata4, Mef2c, and Tbx5 (HNGMT) is >50-fold more efficient than GMT alone, with higher cardiac related gene expression and more cells exhibiting calcium transient and spontaneous beating. Focusing on cardiac fibroblasts isolated from adult mice with myocardial infarction (MICFs), a five factor combination, GMTMS (GMT plus Myocd and Sall4), induces more iCMs expressing the cardiac structural and functional proteins [8].

### 2.2. Transcriptional Factors Optimization

Aside from screening additional transcriptional factors for cardiac reprogramming, there are other findings related to transcriptional factors themselves. The fusion of the powerful transactivation domain (TAD) derived from MyoD (the M3 domain), firstly used in iPSCs, improves the reprogramming efficiency of iPSCs [9,10,11]. Thus, Hirai et al. have applied the same strategy in cardiac reprogramming and show that fused Mef2c with wild-type Gata4, Hand2, and Tbx5 reaches the highest reprogramming efficiency. Additionally, different isoforms of Mef2c also affect the conversion rate of fibroblasts into iCMs [12].

Due to the fact that fibroblasts should be infected by each of the three transgene vectors, a “Triplet” polycistronic vectors encoding Gata4, Mef2c, and Tbx5 which has ensured infected cells overexpressing three transcriptional factors simultaneously is constructed [13]. Subsequently, the stoichiometry of Gata4, Mef2c, and Tbx5 has been found to play a significant role in cardiac reprogramming [14]. After comparing all six possible splicing orders of Gata4, Mef2c, and Tbx5, researchers found that MGT vectors result in a more than 10-fold increase in the number of beating iCMs, which is caused by different protein expression levels of the three transcriptional factors. Similarly, stoichiometric optimization of Gata4, Hand2, Mef2c, and Tbx5 also further enhances the efficiency of cardiac reprogramming [15].

Recently, Zhang et al. indicated that ensuring the expression of all transcription factors enhances cardiac reprogramming [16]. By selectively analyzing subpopulations of reprogrammed cells, they showed that iCMs with all transcriptional factor overexpression possess higher cardiac related gene expression levels and contractile cardiac structure formations. Their research emphasizes the significance of all transcriptional factors’ co-expression in the system, which has provided an important guideline for future studies.

### 2.3. Mechanisms of the Transcriptional Network in Cardiac Reprogramming

Though researchers have worked out numerous strategies for manipulating transcription networks in cardiac reprogramming, the underlying mechanisms remain largely unknown. Therefore, a deep understanding of the conversion process should provide us with new insights and inspiration for future studies and applications.

Gata4, one of three cardiac reprogramming factors, is a master transcriptional factor in heart development. Gata4 has been identified to downregulate pro-fibrotic factors and mediators, including Snai1, during the process of GMT-induced reprogramming [17]. Moreover, Fernandez-Perez et al. have identified the function of Hand2 in the induction of pacemaker-like myocytes (iPMs) [18]. As a subtype of cardiac reprogramming, they find that Hand2 plays a vital role in the combination of GHMT. Hand2 augments chromatin accessibility at loci involved in sarcomere formation, electrical coupling, and membrane depolarization. Selective reorganization of chromatin accessibility by Hand2 provides possibilities for pacemaker specific gene expression, thus completing the reprogramming of iPMs. Further study reveals that all transcription factors in GHMT synergistically activate genome-wide cardiogenic specific enhancers, which has helped us reach a deeper understanding of the transcription network in cardiac reprogramming [19].

Single-cell technology was first reported in 2009, which has provided excellent opportunities to study individual cells in different biological stages [20]. Liu et al. have delineated the antagonistic relationship between cell proliferation and iCM induction by analyzing single-cell transcriptomes during cardiac reprogramming [21]. Intriguingly, they have also revealed the unexpected downregulation of factors involved in mRNA processing and splicing. Focusing on this finding, they have identified several splicing factors as barriers of cardiac reprogramming including Sf3a1, Sf3b1, and Zrsr2 through loss-of-function experiments [22]. Continued studies of the mechanisms of the transcriptional network related to combinations of TFs direct cardiac lineage conversion are required for revealing the nature of the conversion process [23], which may provide more potential targets for future research and clinical translation.

## 3. Epigenetic Regulations in Reprogramming

### 3.1. MicroRNAs

MicroRNAs have been uncovered in directing cardiac reprogramming due to their unique roles in cell fate changes [24]. A screen of candidate miRNAs based on their roles in cardiac muscle development and differentiation has allowed Jayawardena et al. to identify a combination of microRNAs (miRNA) 1, 133, 208, and 499 that can reprogram mouse cardiac fibroblasts into iCMs. In their in vitro study, calcium oscillations are observed in a miRNA treated group but with only a few exhibited spontaneous contraction. Mechanistically, these miRNAs likely target Nanog to induce early cardiac reprogramming. These miRNAs can also directly repress Snai1 and silence pro-fibrotic signatures. The same group also identified that the addition of an RNA-sensing receptor ligand, called ICR2, further enhances the ability of reprogramming factors to produce iCMs by targeting the RNA-sensing receptors Rig-I and TLR3 [25].

Interestingly, miR-1 and miR-133a can also enhance MGT-based reprogramming by silencing pro-fibrotic signatures [3]. Although it has been shown that miR-133a can be used to enhance cardiac reprogramming in human cells, whether the combination of reprogramming factors with miRNAs could further improve heart function and cardiac reprogramming efficiency in vivo, remains to be explored.

### 3.2. Chromatin Remodeling Factors

Chromatin remodeling factors also affect reprogramming. BAF60c, a cardiac-specific subunit of ATP-dependent chromatin remodeling SWI/SNF complexes, can increase cardiac reprogramming with MGT [6], which is consistent with a previous finding that Baf60c can initiate ectopic cardiac gene expression in mouse mesoderm with Gata4 [26,27].

Another chromatin remodeling factor HELLS, has also been identified to increase reprogramming by screening, although the mechanism is unknown [28].

### 3.3. Epigenetic Factors Related to Methylation

The epigenetic factors related to methylation have also been found to regulate cardiac reprogramming. By screening 35 selected components of chromatin modifying or remodeling complexes, researchers have identified the polycomb complex gene Bmi1 as a major barrier for cardiac reprogramming [28]. Bmi1 directly binds to a set of key cardiogenic loci that are co-occupied by other PRC components. Reduced Bmi1 expression corresponds with increased levels of the active histone mark H3K4me3 and reduced levels of repressive H2AK119ub at cardiogenic loci. More importantly, Testa and colleagues showed that the Bmi1 inhibitor PTC-209 promotes the chemically-induced direct cardiac reprogramming of cardiac fibroblasts into cardiomyocytes [29]. Our group has identified H3K4 methyltransferase Mll1 in inhibiting iCM reprogramming based on a screen of 47 cardiac development-related epigenetic and transcription factors [30]. Inhibiting Mll1 activity with small molecules improves efficiency in converting embryonic fibroblasts and cardiac fibroblasts into functional cardiomyocyte-like cells. Since Bmi1 and Mll1 belong to two complexes with opposite functions for methylation, it is rather intriguing that both can enhance reprogramming. More interestingly, both Bmi1 and Mll1 are involved in not only cardiac reprogramming, but also iPSC [31] and pluripotent stem cell reprogramming [32]. These results imply that Bmi1 and Mll1 might have more general targets instead of the specific cardiogenic loci.

Along with methylation writers and erasers, histone readers, such as PHF7, which directly bind to histone H3K4me3 and H3K4me2 marks, also play a significant role in cardiac reprogramming [33,34]. Mechanistically, PHF7 can bind to cardiac super enhancers and increase chromatin accessibility by interacting with the SWI/SNF complex. More interestingly, Phf7 plus Mef2c and Tbx5 can achieve efficient cardiac reprogramming without Gata4, as confirmed by immunocytochemistry, flow cytometry, and quantitative PCR (qPCR).

The current body of work regarding the epigenetic regulation of cardiac reprogramming represents only the tip of the iceberg. Although chromatin remodeling and histone methylation related epigenetic regulation have been studied, very few studies have pursued other epigenetic regulations in cardiac reprogramming, such as histone acetylation and phosphorylation.

## 4. Cellular Microenvironment in Cardiac Reprogramming

### 4.1. Growth Factor Regulation

One earlier investigation showed that pre conditioning infarcted myocardium with vascular endothelial growth factor enhances cardiac reprogramming and heart function [35]. Subsequently, another study showed that a combination of FGF2, FGF10, and VEGF (FFV medium), significantly enhances cardiac reprogramming, including, most importantly, a 100-fold increase in beating iCMs [36]. Notably, these growth factors have also been used in cardiomyocyte differentiation protocols. Moreover, Gata4 is not required when using FFV medium, enabling the induction of functional cardiomyocytes via only Mef2c and Tbx5.

### 4.2. Signaling Pathways

Signaling pathways always lead to the activation of certain kinases. The importance of kinase regulation has been shown in cell fate change. One such study screened a constitutively activated protein kinase library and identified Akt1/protein kinase B as an enhancer for reprogramming [37]. Akt1 induces spontaneous beating in approximately 50% of reprogrammed mouse embryo fibroblasts after 3-week induction with GATA4, HAND2, MEF2C, and Tbx5 (GHMT). Furthermore, the addition of Akt1 to GHMT results in a more mature cardiac phenotype for iCMs [37]. A follow up study indicates that Akt1 acts synergistically with Hand2 to recruit other TFs to enhancer elements [19]. Based on these studies, a cardiac reprogramming gene regulatory network has been established and within this network, repressing EGFR signaling and JAK pathways can enhance reprogramming.

Another important pathway identified to regulate cardiac reprogramming is the TGF beta pathway [38]. Researchers utilized calcium indicator GCaMP driven by the cardiac Troponin T promoter to quantify iCM yield and identified the TGF β inhibitor, SB431542 (SB), as a small molecule capable of increasing the conversion of both mouse embryonic fibroblasts and adult cardiac fibroblasts to iCMs up to 5-fold [38]. It has been revealed that the TGF β pathway is activated during the early stage of reprogramming, whereas overactivation of pro-fibrotic signaling networks by TGF β attenuates cardiac reprogramming [3]. TGF β inhibitor is not the only molecule shown to enhance cardiac reprogramming in vitro. Another study screened 5500 compounds in primary cardiac fibroblasts and found that transforming growth factor-β inhibitor SB431542 and the WNT inhibitor XAV939, increase reprogramming efficiency 8-fold when added to GMT-overexpressing cardiac fibroblasts. More importantly, SB431542 and XAV939 also enhance in vivo reprogramming with GMT [39].

### 4.3. Inflammation Regulation

Our laboratory has identified four agents, insulin-like growth factor-1 (IGF1), Mll1 inhibitor MM589, that transform growth factor-β inhibitor A83-01, and Bmi1 inhibitor PTC-209, termed IMAP, coordinately enhancing reprogramming efficiency. IMAP treatment represses many genes involved in immune responses, particularly those in specific C-C chemokine signaling pathways [40]. Therefore, we investigated the roles of C-C motif chemokine ligand 3 (CCL3), CCL6, and CCL17 in cardiac reprogramming and observed that these ligands inhibit iCM formation, whereas inhibitors of C-C motif chemokine receptor 1 (CCR1), CCR4, and CCR5 have the opposite effect. This is not the only example of the role of inflammation signaling for cardiac reprogramming. Another study screened 8400 chemical compounds and found that diclofenac sodium (diclofenac), a non-steroidal anti-inflammatory drug, greatly enhanced cardiac reprogramming [41]. The major effect of diclofenac is silencing the inflammatory effect by targeting cyclooxygenase-2, prostaglandin E2/prostaglandin E receptor 4, cyclic AMP/protein kinase A, and interleukin 1β signaling. Interestingly, for the microRNA mediated cardiac reprogramming, JAK inhibitor I treatment also enhances reprogramming efficiency [24]. Notably, the JAK-STAT pathway plays an important role in inflammation [42].

Olson and colleagues screened 1052 ORF cDNAs, which led to the discovery of 49 activators and 129 inhibitors of cardiac reprogramming. They found that one of the most potent activators, ZNF281, stimulates cardiac reprogramming by genome-wide association with GATA4 on cardiac enhancers and the recruitment of a NuRD complex. The Nucleosome Remodeling Deacetylase (NuRD) complex is a group of associated proteins related to chromatin remodeling and histone deacetylase activities. The major effect of the NuRD complex is attributed to its anti-inflammatory function, which is consistent with the finding that anti-inflammatory drugs dexamethasone and nabumetone also promote cardiac reprogramming [43]. Interestingly, ZNF281 have important functions in somatic reprogramming and the maintenance of pluripotent states [44,45].

### 4.4. Cellular Matrix Regulation

Mechanical properties represent another important element of the cellular microenvironment. Given that the tissue elasticity of the myocardium is much softer than that of culture dishes, Shotaet al. studied the effect of matrix stiffness on cardiac reprogramming [46]. They found that the soft matrix was comparable with native myocardium, promoting the efficiency and quality of cardiac reprogramming. Mechanistically, this is due to the inhibition of integrin, Rho/ROCK, actomyosin, and YAP/TAZ signaling, as well as the suppression of fibroblast programs. However, as the infarcted myocardium stiffens during the first 1 to 2 weeks [47], studies to specifically understand the effect of mechanical propertiesof cardiac reprogramming in the infarcted heart are required.

In summary, studies of the cellular microenvironment in cardiac reprogramming have identified cardiogenesis signaling (FFV and Akt1), inflammation signaling, pro-fibrotic signaling, and mechanical properties as important regulators for reprogramming. Since cardiac lineage differentiation and immune lineage differentiation are highly related during development [48], inhibition of inflammation signaling during cardiac reprogramming might also reflect the requirement of inhibition immune lineage cells during cardiac lineage development.

## 5. Cell Cycling Regulation in Cardiac Reprogramming

In iPSC reprogramming, increased cellular proliferation through cell cycle regulation improves reprogramming efficiency [49]. However, the role of cell cycle regulation during the progression of iCM reprogramming has not been well defined. The active cell-cycle status at the later stage of reprogramming has been found to be negatively correlated with the maturity of reprogrammed iCMs [21,50], and iCM reprogramming is significantly suppressed in an immortalized cardiac fibroblast line which never exits the cell cycle [21]. Single cell genomics is an effective approach to address the problem of inherent heterogeneity and the asynchronous cell fate switching process in cardiac reprogramming [21,51]. Qian et al. analyzed global transcriptome changes during the early stages of iCM reprogramming by single cell RNA-seq [21], and revealed that decreased cardiac fibroblast proliferation, or cell cycle synchronization, enhances iCM reprogramming, whereas increased proliferation suppresses iCM generation [21]. On the other hand, studies revealed that iCM-reprogramming is predominantly initiated at the late-G1- or S-phase and nearly half of GMT-reprogrammed iCMs divide soon after reprogramming through 48 h time-lapse recordings. S-phase synchronization post-GMT infection enhances the cell cycle arrest of reprogrammed iCMs, yield more iCMs with the expression of cardiac genes, and accelerates the early progression of reprogramming [52], which has also been demonstrated in human somatic cell reprogramming [53]. In the presence of the transforming growth factor (TGF)-β inhibitor SB431542, and the WNT inhibitor XAV939, GO enrichment analysis of the RNA-seq data from 3 weeks reprogrammed iCMs identified that both the cell cycle and M phase of the mitotic cell cycle are downregulated in the accelerated reprogramming process [39]. Hand2 suppresses cell cycle promoting genes and also physically interacts with GMT to activate cardiac gene expression in cardiac reprogramming [54]. Furthermore, the addition of Akt1 to GHMT leads to a quick cell cycle exit and evokes the maturation features of the cardiac phenotype in the transcriptional reprogramming of fibroblasts to functional cardiomyocytes [37]. However, some opposing views still exist. P27, which is known to be involved in cell cycle arrest and cell differentiation, appears unnecessary for Notch inhibition DAPT enhanced GHMT-induced cardiac reprogramming [55]. MEF2C, as an important transcription factor in cardiac direct reprogramming, plays an antagonistic role to that of MEF2A and MEF2D by activating cell cycle genes and repressing markers of terminal differentiation [56].

## 6. Clinical Translational Progress for Cardiac Reprogramming

### 6.1. Non-Exogenous Genes Induced Cardiac Reprogramming

Due to the limitation of gene therapy, several strategies have been used to advance cardiac reprogramming closer to clinical application. Sequentially replaced exogenous genes with chemicals have proven successful in iPSC reprogramming field [57,58]. It appears that GATA4 is dispensable by shRNA knockdown of Bmi1 [28], FFV medium [36], and several other strategies. However, whether is it possible to further replace Mef2c and Tbx5 with chemicals is unknown.

Pure chemically induced reprogramming is also an attractive strategy. Using a small molecule combination (CRFVPTZ (C, CHIR99021; R, RepSox; F, Forskolin; V, VPA; P, Parnate; T, TTNPB; and Z, DZnep)), beating CMs can be induced from mouse fibroblasts [59]. Human fibroblasts can also be induced into CMs with nine small molecules (CHIR99021, A83-01, BIX01294, AS8351, SC1, Y27632, OAC2, SU16F and JNJ10198409) [60]. In addition, GSK3 inhibitor (CHIR99021), TGF β inhibitors (A83-01, SB431542 and RepSox) and Wnt/β-catenin inhibitor (XAV939) should be emphasized for their potential clinical application in cardiac reprogramming [3,39]. However, strategies to minimize the requirement of chemical combination, and the accurate targeting of cardiac fibroblast with chemicals, need to be further studied.

Compared with viruses, mRNA has its potential advantages including higher transfection efficiency and weaker innate immune responses. Thus, Lee et al. have developed a strategy named peptide-enhanced mRNA transfection that appears safer and more efficient than original viral infection methods [61]. Moreover, Kim et al. have optimized the mRNA transfection process with deoxycholic acid-conjugated PEI (DAPEI) that acts as the most efficient non-viral gene delivery carrier among several commercial and synthetic non-viral vectors [62].

Similar to transcriptional factors, miRNAs have also been investigated in vivo for their heart regenerative function. Jayawardena et al. applied a miR combo (miRs-1, 133, 208, 499) into ischemic mouse myocardium and observed direct reprogramming from fibroblasts into iCMs in situ. Genetic tracing analysis indicates that these induced cells are most likely of fibroblastic origin. Subsequent studies explored the biological and functional consequences of such reprogramming [63]. FS is partially recovered in the miR combo group, while fibrosis is significantly decreased simultaneously 2 or 3 months after treatment. Thus, miRNAs provide another alternative for future cardiac reprogramming in clinical applications.

### 6.2. Viral Gene Based Delivery System Optimization

In the first report, Ieda et al. transplanted cardiac fibroblasts 1 day after transfection with GMT into mouse hearts [2]. They observed that transplanted cells express α-actinin and develop sarcomeric structures 2 weeks after transplantation. However, the potential cardiac regenerative or protective function caused by reprogrammed cell transplantation was not described. Later, Qian et al. from the same group applied the in vivo delivery of GMT into mouse hearts with myocardial infarction [64] and observed decreased infarct size and improved cardiac function. Specifically, eject fraction (EF) raises from 20 to 30% 4 weeks after GMT infection. Similarly, GHMT was also tested in the same mouse model [5] and GHMT treatment blunts the worsening of EF and reduces adverse ventricular remodeling in both short-term (3 weeks) and long-term (12 weeks) experiments.

In most studies, transcriptional factors would be integrated into the genome of hosts by lentiviruses or retroviruses, which is a significant drawback of this approach. Therefore, a safer delivery system is required for future studies and clinical use. Different from lentiviruses or retroviruses, the application of adenoviruses was nonintegrating, which was genetically safer in usage. According to Mathison et al., adenoviruses can be used for direct cardiac reprogramming in vivo experiments, and achieve a similar heart protection effect to a lentivirus-based system, which improves ejection fraction when compared with the control group (AdGMT, 21 ± 3% vs. LentiGMT, 14 ± 5%) [65].

Intriguingly, GMT delivery by another nonintegrating virus, Sendai virus (SeV), results in 100-fold more beating iCMs than retroviral-GMT and shortens the time to induction of beating cells from 3 to 10 days in mouse fibroblasts [66]. SeV-GMT appears to be more efficient than the retroviral vector in both mouse and human cardiac reprogramming, suggesting its potential for future heart regenerative studies. Follow-up study also confirms that SeV-MGT induces iCMs in MI mouse model with lineage tracing [67] and SeV-GMT treatment significantly improves fractional shortening (FS) compared to pMX-MGT after 4 weeks which indicates its superior cardiac protection effect.

### 6.3. Non-Viral Gene Based Delivery System Optimization

Nanocarrier is a promising tool for gene and drug delivery. Beside the early studies mention above concerning the successful delivery of mRNA and microRNA in vitro, there are also nanocarriers developed for in vivo cardiac reprogramming that have been shown to have a therapeutic potential. Lei et al. designed branched polyethyleneimine modified nitrogen-enriched carbon dots nanocarriers to load the miRNAs-combo (miRNA-1, miRNA-133, miRNA-208, and miRNA-499). These nano complexes led to master cardiac transcription factor activation and efficient direct reprogramming of fibroblasts into iCMs. More importantly, the nano complexes treatment group significantly decreased the fibrotic area and infarct thickness in a mice myocardial infarction model. The authors also showed that the nanocomplexes increase cardiac regeneration as evidenced by the increased CD31 signal [68]. Qiaoziet al. developed nanoparticles that can specifically target cardiac fibroblasts via mimicking neutrophils and homing into the injured heart after MI [69]. By coating FH peptide-modified neutrophil-mimicking membranes on mesoporous silicon nanoparticles (MSNs), loaded with microRNA 1, 133, 208, and 499 (miR Combo), intravenous injection of the nanoparticles successfully delivered miR Combo into fibroblasts and led to efficient reprogramming, resulting in improved cardiac function and attenuated fibrosis.

Besides the delivery of microRNA, Yujunget al. showed that cationic gold nanoparticles (AuNPs) can load Gata4, Mef2c, and Tbx5 expression plasmids for cardiac reprogramming [70]. The AuNP/GMT/PEI nano complexes show promising reprogramming efficiency in vitro and result in the effective recovery of cardiac function and reduced scar area in vivo.

In addition to the delivery of basic MGT or miRNAs combinations, it would be more exciting to develop nanocarriers to more efficiently and simultaneously deliver exogenous genes and chemicals. Fabianaet al. proposed that an enhanced permeability and retention (EPR) effect can be taken advantage of in nanocarrier-based gene and chemical delivery [71]. Although the effect of EPR in myocardial infarction has not been well studied, the effect of EPR in other ischemia models has been accessed. Christopheret al. showed that in a murine model of hind limb ischemia, 64 Cu-labeled PEGylated reduced graphene oxide—iron oxide nanoparticles (64Cu-RGO-IONP-PEG) displayed substantial accumulationin the ischemic tissue [72]. Specifically developed nanocarriers can target ischemic hearts by passive targeting or combined with active targeting, resulting in the improvement of cardiac reprogramming for clinical applications.

### 6.4. Reprogramming Human Fibroblasts into iCMs

Compared with mouse cells, human somatic cells are more resistant to lineage conversion. According to Nam et al., GMT can barely reprogram human foreskin fibroblasts into iCMs with a less than one percentage of cTnT+ cells [73]. Therefore, additional transcriptional factors, Hand2 and myocardin, are identified and along side GMT (GHMMyT), these five factors show a greater CM-inducing effect in human fibroblasts conversion. A similar screening assay reveals that GMT, Mesp1, and Myocd (GMTMM) also elevate cardiac-specific gene and protein expression levels [74]. Furthermore, GMTMM induced iCMs exhibit action potentials and contraction when cocultured with murine cardiomyocytes. A third group shows that GMT plus ESRRG and MESP1 also directly reprogram human fibroblasts into iCMs [75]. Introducing Myocardin and ZFPM2 further improves reprogramming efficiency including global cardiac gene expression, sarcomere formation, and calcium transients. Apart from transcriptional factors, gene delivery using carboxymethylcellulose (CMC) nanoparticles (CiCMC-NPs) and specific drug 5-azacytidine (5-AZA) that inhibits cell proliferation, has also been applied in human fibroblast conversion [76]. Nevertheless, the conversion rate still remains low with human cells compared with murine cells.

Similarly, researchers have also investigated whether miRNAs played a significant role in human cardiac reprogramming and attempted to optimize the miRNA strategy. The group who reported GHMMyT found that two muscle-specific miRNAs, miR-1 and miR-133, can exert reprogramming activities and enhance human cardiac reprogramming, working synergistically with GHMMyT [73]. Moreover, Bektik et al. performed a single-cell quantitative PCR, showing that miR-1 and HAND2 further improve 7-factor-mediated human iCM reprogramming [51]. MiR-133, which promotes mouse cardiac reprogramming, also exerts great enhancement on human cardiac reprogramming by the same mechanisms and stimulates more spontaneous beating cells compared to the transcriptional factors only group [77]. Christoforou et al. applied this combinatorial core transcriptional factor and microRNA strategy to globally change the expression profile during reprogramming [78]. Moreover, after introducing GATA4, TBX5, MEF2C, MYOCD, and NKX2-5, together with miR-1 and miR-133a, human fibroblasts partially organize their cytoskeleton in a cross-striated manner and exhibit active calcium transients. However, spontaneous contractions remain to be accomplished.

Due to low reprogramming efficiency caused by unclear barriers, Yang Zhou et al. performed single-cell RNA sequencing in human cell cardiac reprogramming which allows for the development of a cell fate index of the whole biological process [79]. This index has provided detailed molecular features that may guide future studies to further enhance cardiac reprogramming.

## 7. Conclusions

In summary, cardiac reprogramming is becoming a promising therapeutic strategy in heart regenerative medicine. We anticipate continued progress in basic mechanistic and preclinical studies. We also expect that in addition to the optimization of the cardiogenesis related transcriptional regulation and signaling pathway, more research will focus on modulating the pathological cellular microenvironment associated with heart injury including inflammation, pro-fibrotic signaling pathways, and mechanical properties of the damaged myocardium, to facilitate cardiac reprogramming and regeneration in vivo. Non-integrated delivery strategies, such as nanoparticle, mRNA or protein mediated reprogramming, will also be thoroughly investigated in preclinical and clinical studies.

## Data Availability

The GA is created with BioRender.com.
